# Emphysematous Pyelonephritis, Emphysematous Cystitis, and Emphysematous Ureteritis: A Case Report

**DOI:** 10.7759/cureus.29651

**Published:** 2022-09-27

**Authors:** Jaime E Campos, Phia A Martinez, Umme Salma Rangwala, Wolfut Fazli, Carlos Rey

**Affiliations:** 1 Infectious Disease, Florida International University, Herbert Wertheim College of Medicine, Miami, USA; 2 Internal Medicine, American University of Antigua, St. John's, ATG; 3 Internal Medicine, Mahatma Gandhi Memorial Medical College, Indore, IND; 4 Radiology, Florida International University, Herbert Wertheim College of Medicine, Miami, USA

**Keywords:** ureteritis, emphysema cystitis, esbl e.coli, cystitis, pyelonephritis, nephritis, emphysematous

## Abstract

Emphysematous urinary tract infections (EUTIs) are rare, severe, and suppurative infections affecting various parts of the urinary tract. We report a case of a 75-year-old male presenting with hematuria and generalized weakness with uncontrolled diabetes mellitus (DM) and hypertension. He tested positive for COVID-19 on the second day of hospital admission. A non-contrast-enhanced CT of the abdomen and pelvis revealed gas within the left renal parenchyma, walls of the left ureter, and urinary bladder, establishing the diagnosis of EUTIs. The patient was treated using intravenous antibiotics without any surgical intervention, and four weeks later was stable and transported to long-term acute care (LTAC) facility. DM is the most common risk factor for the development of EUTIs and *Escherichia coli* is the most common causative pathogen.

## Introduction

Emphysematous pyelonephritis (EPN), emphysematous cystitis (EC), and emphysematous ureteritis (EU) are defined as infections of acute, severe, necrotizing natures resulting in the accumulation of gas within the renal parenchyma, collecting system, the peri-nephric space, and the wall and lumen of the urinary tract [1]. Kelly and MacCallum reported the first case of emphysematous pyelonephritis in 1893 [2]. To better emphasize the relationship between gas formation and an infectious pathology, Schultz, and Klorfein, in 1962 suggested the use of the term ‘emphysematous pyelonephritis’ [3]. The clinical presentation of these emphysematous urinary tract infections (EUTIs) ranges from asymptomatic infections, pneumaturia, dysuria, fever, and abdominal pain to septic shock, which is also the primary cause of mortality in patients with EPN. The concurrence of EUTIs in cases of EC is 15.4%. EUTIs are life-threatening illnesses with a mortality rate of up to 50% [4-6]. Diabetes mellitus (DM) is the single most common risk factor with 95% of patients diagnosed with EUTIs having uncontrolled DM [5]. Pathogens implicated in the causation of EUTIs include *Escherichia coli*, which remains the most common, and other anaerobes such as *Klebsiella spp.* and *Proteus spp.,* which ferment glucose resulting in necrosis and the formation of gas [7]. Our case report illustrates how the timely institution of an intravenous antibiotic therapy, which is different from what is traditionally used to manage cases of EUTIs, prolonged the survival of our patient despite being afflicted with several comorbidities including a COVID-19 infection acquired while admitted to the hospital.

## Case presentation

A 75-year-old male was brought to the emergency room from an assisted living facility (ALF) with chief complaints of hematuria and generalized weakness for three days. The ALF staff reported a subjective fever, without any other associated signs or symptoms. Past medical history is significant for uncontrolled DM, morbid obesity (BMI >30kg/m^2^), hypertension, hyperlipidemia, hypothyroidism, inferior vena cava (IVC) filter placement, and psychiatric illness of schizophrenia. On the second day of admission, the patient developed atrial fibrillation with a rapid ventricular response (RVR), for which advanced cardiovascular life support (ACLS) protocol was activated immediately, and return of spontaneous circulation (ROSC) was achieved. The patient was intubated and transferred to the critical care unit (CCU) where vasopressor support and mechanical ventilation were initiated. The patient had a negative reverse transcription-polymerase chain reaction (RT-PCR) for COVID-19 on the day of admission, which upon subsequent testing came back positive on the second day of admission; chest X-ray revealed bilateral multifocal ground glass opacities with decreased aeration in both lungs.

**Figure 1:**
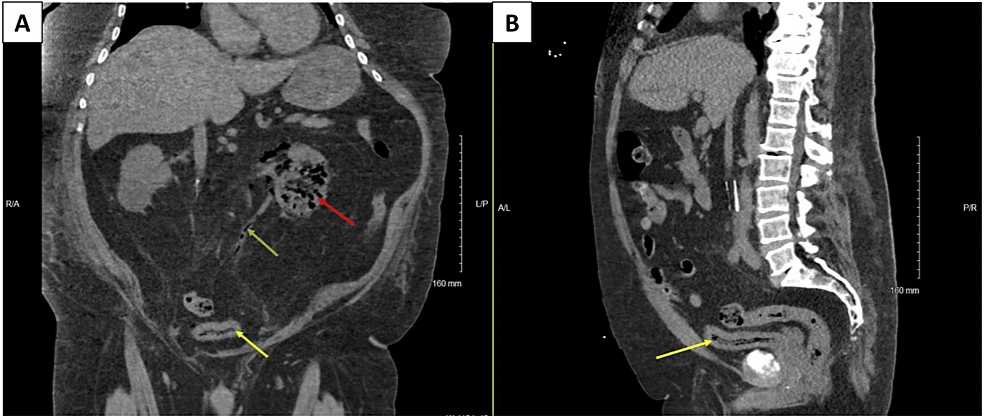
Non-contrast enhanced CT of the abdomen and pelvis Panel A: Coronal view showing gas within the left renal parenchyma (red arrow), wall of the left ureter (green arrow), and wall of the urinary bladder (yellow arrow). Panel B: Sagittal view showing gas within the urinary bladder (yellow arrow).

A non-contrast CT scan of the abdomen and pelvis revealed extensive air accumulation within the left renal parenchyma, walls of the left ureter, and urinary bladder; establishing the diagnosis of emphysematous pyelonephritis, cystitis, and ureteritis (Figure 1). The grading of the EPN according to the Huang and Tseng [8] classification was grade 3B. EUTIs were complicated by septic shock, multiorgan failure, thrombocytopenia, and acute respiratory distress syndrome (ARDS) leading to respiratory failure dependent on mechanical ventilation. During the patient’s hospital admission, he was put on dialysis, a percutaneous endoscopic gastrostomy (PEG) tube was inserted for nutritional support and the urinary bladder was catheterized. Vitals upon admission were as follows: temperature - 98.0 F (36.7 degrees Celsius), heart rate - 125 beats/minute, blood pressure - 130/100 mmHg, and respiratory rate - 21 breaths/minute. The patient was given supplementary oxygen with fraction of inspired oxygen (FiO2) of 100%, which was gradually reduced to 40% over the course of hospital admission. Table 1 lists lab values at the time of admission.

**Table 1:** Laboratory values at the time of admission PaCO2=partial pressure of carbon dioxide; PaO2=partial pressure of oxygen; HCO3=bicarbonate; hpf=high power field; cfu=colony forming units

Complete Blood count (CBC)	
White blood cell (WBC) count	17900/cu.mm
Red blood cell (RBC) count	3.77x10^(6)^/cu.mm
Hemoglobin	11.7g/dL
Platelet count	68000/cu.mm
Differential leukocyte count (DLC)	
Neutrophil	95.1%
Immature granulocyte rel.	6.3%
Lymphocyte	2.3%
Eosinophil	0.1%
Basophil	0.3%
Monocyte	3.9%
Serum	
Sodium	121 mg/dL
Potassium	4.4 mg/dL
Calcium	9.7 mg/dL
Glucose	473 mg/dL
Blood urea nitrogen (BUN)	56.0 mg/dL
Creatinine	3.7 mg/dL
Procalcitonin	0.68 ng/mL
Lactic acid	0.9 mg/dL
C-reactive protein (CRP)	28.9 mg/dL
Arterial Blood Gas (ABG)	
pH	7.167
PaCO_2_	47.2 mmHg
PaO_2_	368.8 mmHg
HCO_3_^-^	16.7 mg/dL
Base excess	-11.7 mEq/L
Urinalysis	
Colour and appearance	Red and turbid
Specific gravity	1.008
pH	5.0
Protein	100 mg/dl
Glucose	>500mg/dl
WBC	>182/hpf
RBC	>182/hpf
Urine culture	70,000 cfu/ml extended-spectrum beta-lactamases (ESBLs) producing *Escherichia coli* (*E. coli*)

**Figure 2:**
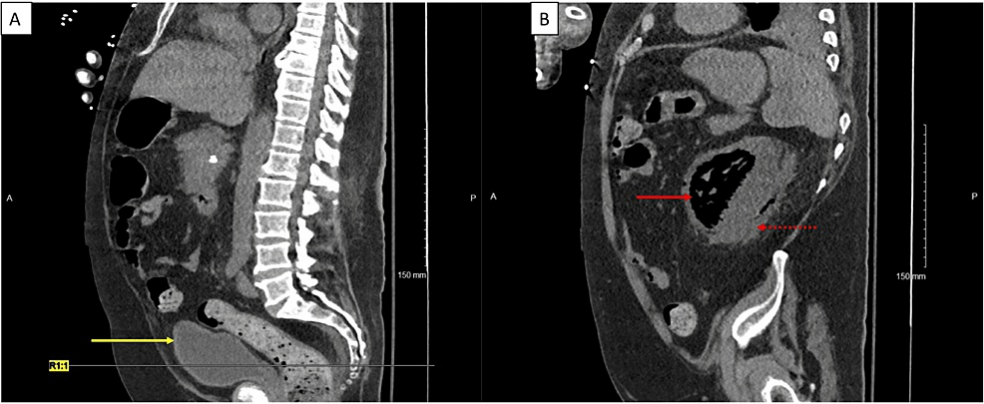
Non-contrast CT scan of abdomen and pelvis Panel A: Sagittal view showing fluid (urine) within the urinary bladder cavity and absence of gas within the wall of the urinary bladder indicating resolution of emphysematous cystitis (EC) (yellow arrow). Panel B: Sagittal view showing gas within the left renal parenchyma (solid red arrow), free fluid in the perinephric space (dotted red arrow), indicating persistence of emphysematous pyelonephritis (EPN).

Our course of management consisting of careful intravenous fluid and pressor management, as well as doses of intravenous meropenem, resulted in the resolution of the EU and EC, as evidenced by a non-contrast-enhanced CT scan done four weeks after treatment (Figure 2A). The EPN, however, could not be resolved as it was of a severe grade (3B according to the Huang and Tseng classification [8]) to begin with and our patient was not a candidate for surgical intervention (Figure 2B). The patient achieved clinical stability four weeks after admission and was discharged to the long-term acute care (LTAC) facility.

## Discussion

EUTI is an umbrella term that encompasses pathologies such as infections of the lower (urethra, bladder, and or prostate) or upper (kidney and ureters) urinary tract associated with gas formation. Acute severe necrosis of the renal parenchyma and surrounding space can also set in the face of delayed management of the disease.

Clinical signs and symptoms vary depending on the extent of involvement of the urinary tract, which more often than not are non-specific, such as fever, non-localized abdominal pain, flank or back pain, nausea, vomiting, altered mental status, and evidence of septic shock. Less common signs include subcutaneous emphysema, pneumomediastinum, and septic embolization of internal organs such as the brain, liver, and lungs [9]. Crepitation over the flank areas is an uncommon clinical sign; if present, it should raise concern for EPN [10]. In the case of suspected EC, the presence of pneumaturia which is the passage of gas during micturition should raise a high degree of suspicion for EC [11]. However, there are no clinical signs or symptoms that are characteristic of EUTIs; therefore, radiological studies should be made use of to quickly establish a diagnosis, thereby expediting the initiation of treatment as EUTIs are life-threatening with a high mortality rate of 70-90% [12,13].

DM is a major risk factor for these infections, of which our patient has a long-standing history [14]. Other noteworthy risk factors include older age group, female gender, neurogenic bladder, bladder outlet obstruction, excessive alcohol consumption, indwelling foley catheters, recurrent urinary tract infections, and immunosuppression [4]. Females are at increased risk of developing UTIs, and this is thought to be the reason for gender predilection in the case of EUTIs as well [5]. In our case report, the patient suffered from an extended-spectrum beta-lactamase (ESBL) *Escherichia coli* infection, which is the most common EUTI-causing bacteria [7]. Other pathogens reported having caused EUTIs include* Proteus mirabilis, Klebsiella pneumoniae*, Group D *Streptococcus*, coagulase-negative *Staphylococcus*, anaerobic microorganisms including *Clostridium septicum*, *Candida albicans, Cryptococcus neoformans and Pneumocystis jiroveci* [5].

The accumulation of gas within the urinary tract can be attributed to three main sources, namely history of instrumentation, fistula formation, and the presence of bacterial infection [10]. Several studies hypothesize the possible contributors to EUTI development. Immunocompromised state, impaired blood flow to tissue and high glucose concentration within tissues, and urinary tract obstruction are thought to be a few important factors. The strongest risk factor for EUTIs, uncontrolled DM, alters the tissue environment within the renal parenchyma by causing ischemia and necrosis, which encourages the growth of microbes that ferment sugar and emanate gas. The physiologically hypoxic state of the renal medulla contributes as well [9].

CT scan is the diagnostic method of choice for EUTIs as it aids in the localization, visualization, and assessment of the extent and severity of the disease [10]. Several modalities of treatment are used to manage cases of EUTIs; however, prompt initiation of antibiotics mainly targeting gram-negative bacteria and percutaneous drainage (PCD) is of utmost importance [7]. The use of emergency nephrectomies for the management of EUTIs is being cast aside in favor of more nephron-sparing approaches such as PCD and stenting of the urinary tract. This shift in treatment strategies is aided by the widespread use of computed tomography (CT) scans for relatively earlier diagnosis of EUTIs, and various advances related to the management of sepsis-associated complications. Delayed nephrectomies may still be performed in cases of a non-functional kidney. Classes of antibiotics used currently as the first line for the management of cases of EUTIs include aminoglycosides, third-generation cephalosporins, and fluoroquinolones [15]. For our patient, intravenous antibiotic therapy consisting of meropenem was used without any surgical intervention.

Even though surgical management of severe cases of EPN is the mainstay, our patient was not a fit enough candidate for this intervention due to his history of chronic, uncontrolled co-morbidities, and critical state.

## Conclusions

We are presenting this case because there is a limited amount of literature describing a patient with thrombocytopenia, septic shock with multiorgan failure, ventilator-dependent respiratory failure, and ARDS being resolved with medical therapy alone. Even though the guidelines recommend the use of interventions such as nephrostomy and nephrectomy, we had to individualize the course of treatment for our patient as he was not a candidate for these surgical interventions. Appropriate management with fluids and vasopressors should also be considered. These findings will offer physicians an alternative, non-invasive treatment approach for patients with EPN, EC, and EU.
